# Improvement of episodic memory retention by a memory reactivation intervention across the lifespan: from younger adults to amnesic patients

**DOI:** 10.1038/s41398-022-01915-z

**Published:** 2022-04-05

**Authors:** Rodrigo S. Fernández, Soledad Picco, Juan Cruz Beron, Luz Bavassi, Jorge Campos, Ricardo F. Allegri, María E. Pedreira

**Affiliations:** 1 Instituto de Fisiología, Biología Molecular y Neurociencias (IFIBYNE - CONICET), Ciudad de Buenos Aires, Buenos Aires, Argentina; 2 Facultad de Ciencias Exactas y Naturales Universidad de Buenos Aires, Ciudad de Buenos Aires, Buenos Aires, Argentina; 3 Department of Cognitive Neurology, Neuropsychiatry and Neuropsychology, Fleni, Buenos Aires, Argentina

**Keywords:** Human behaviour, Psychiatric disorders, Long-term memory

## Abstract

Spontaneous reactivation of recently acquired memories is a fundamental mechanism of memory stabilization. Re-exposure to specific learned cues during sleep or awake states, namely targeted memory reactivation, has been shown to improve memory retention at long delays. Manipulation of memory reactivation could have potential clinical value in populations with memory deficits or cognitive decline. However, no previous study investigated a target memory reactivation approach on those populations. Here we tested the hypothesis that a reactivation-based intervention would improve episodic memory performance in healthy adults and amnestic patients. On Day 1, young adults, old adults and amnestic Mild Cognitive Impairment patients (*n* = 150) learned face-name pairs and 24 h later either received a reactivation intervention or a reactivation control (Day 2). On Day 3, associative and item memory were assessed. A robust Bayesian Generalized Mixed Model was implemented to estimate intervention effects on groups. Groups that underwent the reactivation-based intervention showed improved associative memory retention. Notably, amnestic patients benefited more from the intervention as they also had better item memory retention than controls. These findings support memory reactivation as stabilization and strengthening mechanism irrespectively of age and cognitive status, and provides proof-of-concept evidence that reactivation-based interventions could be implemented in the treatment and rehabilitation of populations with memory deficits.

## Introduction

Normal ageing is associated with cognitive decline and episodic memory impairments related to acquisition and retrieval of previously experienced events [1]. Such diminution is often more pronounced than expected. Mild Cognitive Impairment (MCI or Mild Neurocognitive Disorder) is an intermediate state between normal ageing and dementia [2]. MCI defines a condition of cognitive deficits associated with an objective memory impairment without compromising everyday functioning. In particular, episodic memory impairment is the hallmark of the amnesic MCI subtype (aMCI). The prevalence of MCI among individuals >60 years is ~6.7–25.2% and the progression to neurodegenerative conditions is estimated to be between 5 and 17% [2, 3]. Due to ageing population, cognitive decline and dementia are considered global challenges for health and social care systems.

Treatment options aim to improve cognitive functions and prevent or delay progression from MCI to dementia [3, 4]. In this sense, current pharmacological and cognitive interventions have limited or modest success [5–7]. In particular, cognitive interventions such as cognitive stimulation or rehabilitation use learning and memory strategies as a part of a multicomponent treatment, where the effectiveness of a technique in isolation is difficult to assess. In line with traditional memory frameworks, several compensatory and instructional techniques are applied to improve memory acquisition and retrieval (i.e., visual imagery, external aids, spaced retrieval, errorless learning, etc.; [6–8]). However, there is no linear relation between the amount of information acquired and its subsequent behavioral output (retrieval). Memories are not carved in stone. Events surrounding memory acquisition or retrieval change the process of memory stabilization and its properties [9]. Post-encoding processes gradually stabilize a newly formed representation by the process of memory consolidation [10, 11]. Previous studies found that aMCI patients have memory consolidation deficits. Patients with aMCI are thought to have memory impairments when tested at long delays after acquisition and a higher susceptibility to memory interference [12–16]. This suggests that information loss after memory consolidation is increased in aMCI patients relative to healthy older adults and that the time interval between memory acquisition and retrieval is critical for memory maintenance.

Evidence from awake and sleep studies indicate that shortly after learning, activity patterns presented during memory acquisition are activated again strongly [17–19]. This spontaneous reactivation of recently acquired representations is thought as the mechanism underlying memory stabilization and maintenance [18–20]. Sequential reactivation of hippocampal ensembles along hippocampal-cortical interactions promotes memory stabilization [21, 22]. Studies targeting memory reactivation during sleep or focusing on awake reactivation showed that this process could strengthen, weaken, or change memory shortly after memory acquisition [23, 24]. Typically, the presentation of cues associated with the to-be-remembered information during slow-wave sleep, improves memory stabilization and retention. Similar findings come from the reconsolidation framework, which implies longer time frames to study memory changes. Briefly, reconsolidation is a memory process by which reactivated long-term memories are transiently destabilize, followed by its re-stabilization to update their strength or content [25, 26]. Multiple studies demonstrated that the presentation of specific reminders (reactivation) of information learned one or several days before improves memory precision, retention and protects it against interference [27–29]. However, targeted memory reactivation and stabilization processes in older adults were scarcely studied and with mixed results. In addition, reactivation-based benefits during sleep seem to decline as a result of normal ageing [30]. For example, using a targeted memory reactivation protocol during sleep in older adults, Cordi and colleagues [31] found no benefit on vocabulary. In contrast, Johnson et al. [32] showed that memory reactivation during a nap enhances skill performance. Other studies on episodic memory reconsolidation indicated that memory reactivation strengthens the performance of older adults when tested two or seven days later [33, 34]. Similar results were found using transcranial Direct Current Stimulation [35]. No previous study has examined memory targeted reactivation in populations with memory declines such as aMCI or dementia.

There is a need for improvement in the strategies targeting memory decline and the treatment of aMCI. Considering that older adults and aMCI patients have memory consolidation impairments, it would be beneficial to develop interventions based on the time interval between memory acquisition and retrieval in order to improve episodic memory retention and maintenance.

Thus, this proof-of-concept study tested the hypothesis that a reactivation-based intervention on a stabilized memory would improve episodic memory retention irrespectively of age and cognitive status. For addressing this hypothesis, young adults, healthy older adults, and aMCI patients were trained in face-name pairs (Day 1) and 24 h later received a reactivation intervention or a reactivation control (Day 2). Final memory performance was assessed 24 h later (Day 3). To measure the specific effect of memory reactivation on memory retention, participants were first tested on associative memory (face-name pairs) and then on item memory (faces/names separately).

To our knowledge, this is the first attempt to investigate the potential therapeutic utility of a reactivation-based intervention targeting the time interval between memory acquisition and retrieval in a population with objective memory impairments. In addition, we selected an associative memory task that reflect a common cognitive complaint in the elderly (face-name association [36]). Finally, this work makes a step forward contributing to develop new treatment strategies and targets.

## Methods and materials

### Participants

A total of 150 individuals participated in the study. Sample size was based on a power analysis of our previous memory strengthening studies [27, 28] and Monte Carlo simulations using the R package *simr* targeting the interaction between group and block (minimum effect size of 0.5 with 80% power, with confidence intervals above the 95% level). Young adults (*n* = 50) with no history of neuropsychiatric disorders (age *M* = 24.6, SD = 3.1, 62% females) were recruited via social media and at the University of Buenos Aires campus. Healthy older adults (*n* = 50, age *M* = 73.2, SD = 4.9, 58% females) and aMCI patients (*n* = 50, age *M* = 72.6, SD = 5.3, 50% females) were matched for age and education. Older participants were recruited from the Department of Cognitive Neurology, Neuropsychiatry, and Neuropsychology at Fleni and underwent clinical evaluation, including a neuropsychological test battery (Supplementary Materials S1). () which included: Mini Mental State Examination (MMSE), Logical memory test from the Weschler Memory Scale III, Boston Naming Test, Categorical and Phonological Verbal Fluency Test, Digit Span Forward and Backward, Trail Making Test A and B, Rey Auditory Verbal Learning (RAVLT), Rey–Osterrieth Complex Figure and the Digit Symbol-Coding subtest of the Weschler Adult Intelligence Scale-IV. Diagnosis was based on consensus by a team of neurologists and neuropsychologists following standard guidelines. Before the experiments, participants signed a written informed consent form approved by the Ethics Committee of Fleni.

### Stimuli

As older adults have demonstrated an associative impairment in learning face-name pairs [36], we paired ten neutral faces with ten names (five males and five females, respectively). Faces from older adults were selected from the FACES database [37]. Additional ten faces were used during the item memory recognition. Common names from 1930–1955 were drawn from the civil government registry (https://nombres.datos.gob.ar/) in order to ensure name familiarity in older adults. All names had three syllables and started with a different one. Experimental tasks were designed and presented using MATLAB 2016 (Mathworks Inc., Sherborn, MA, USA) with the Psychtoolbox toolkit.

### Procedure

**Fig. 1:**
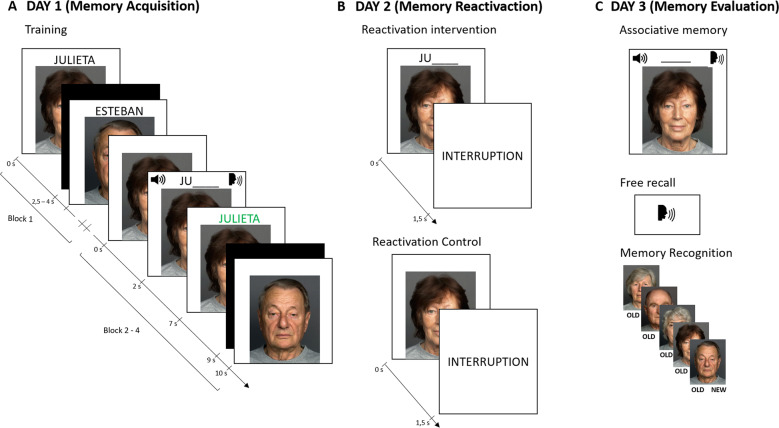
Experimental design. **A** On day 1, participants (*n* = 150) underwent training to learn ten face-name associations. On the first block, the presentation of each face was followed by the presentation of the complete name. Then, for each pair, the face was presented first followed by the presentation of a sound cue, a “speak” legend plus the first syllable of the name. Participants responded aloud the complete name only after the sound cue and “speak” legend presentation. Each response was always followed by feedback on screen. **B** On day 2, groups received either a reactivation intervention or a reactivation control. In the reactivation intervention, each face was presented with the first name syllable followed by an interruption message. Conversely, in the reactivation control, each face was presented alone. In both cases, participants were not allowed to respond as neither the sound cue nor the “speak” legend appeared. **C** On day 3, participants performed three testing sessions: (1) associative memory (face-name pairs): each face was presented alone and participants were instructed to respond aloud the complete name; (2) free recall (item memory): subjects were instructed to say aloud, all the names that they could recall; and (3) memory recognition (item memory): participants performed an old/new task in which they were instructed to decide if the presented face was previously learned (old) or not (new).

As memory stabilization is thought of as a gradient, recently acquired memories are fragile and susceptible to disruption [11, 25]. In contrast, more stabilized memories are more time and interference-resistant. Hence, changes in memory stability and strength would require time to be detected. In consequence, we designed a 3-day study (Fig. 1) with a 24 h interval between sessions as follows:

#### Day 1 (memory acquisition)

Participants were trained in a face-name association task divided into four blocks. On the first block, the ten face-name pairs were presented sequentially and randomly on the center of the screen for 4 s. On blocks 2–4 (tr1 to tr3), faces were first presented alone for 2 s and then the name´s first syllable appeared on the top of the screen along with a sound cue (1 s) with a “speak” legend which signaled that the participant was allowed to respond. Subjects responded aloud the complete name of the face and always received feedback for 2 s. Feedback consisted of the automatic presentation of the correct answer onscreen in a specific color (green). Participants had 4 s to respond and were instructed that they could respond only after the sound cue with the “speak” legend. All answers were recorded with the computer within the task. The inter-stimulus interval on every trial varied between 2.5 and 4 s and the entire procedure took 15 min. All participants reached the inclusion criteria (≥60% of correct responses on the last block).

#### Day 2 (memory reactivation)

Younger adults, older adults, and aMCI patients were randomly assigned either to a reactivation intervention or a reactivation control. Participants were instructed to perform the same face-name task again as on Day 1 and to remember that they could only respond when they heard the sound cue and the “speak” legend.

##### Reactivation intervention

Based on our previous work and sleep studies [20, 27, 28, 38], we constructed incomplete reminders in order to reactivate the stabilized memory acquired on Day 1. This type of reactivation session was demonstrated to have superior effects on episodic memory retention than other types of reactivation. The reactivation session consisted of the presentation of each face for 2 s followed by the name´s first syllable for 1.5 s and an interruption message (“*Trial interrupted*”). Participants were not allowed to respond as neither the sound cue nor the “speak” legend were presented. Correct answers were not presented either. Two reactivation rounds for each face were used, and on each round the presentation order was randomized. The inter-stimulus interval was identical to Day 1.

##### Reactivation control

The procedure for the reactivation control was equivalent to the reactivation intervention, with the exception that the name´s first syllable was not presented. This type of intervention is thought to have a minimal effect on memory performance [26–28]. As not every memory reactivation is capable of improveing memory retention, we used this intervention to control the reactivation itself and explore the specificity of the reactivation intervention. Item and inter-item duration were the same as on the reactivation intervention.

#### Day 3 (memory evaluation)

Our primary interest was to assess memory reactivation effects on associative memory (face-name pairs). However, because associative and item memory may correspond to different ways to retrieve or represent information [39], we also tested item memory by employing free recall (names alone) and recognition (faces alone) tasks. All participants performed memory evaluation in the same order with a 5 min break: first associative memory, then free recall, and finally memory recognition testing.

##### Associative memory (face-name pairs)

Memory retention was assessed across four blocks (ts1 to ts4). Each face was presented on screen and participants were instructed to respond aloud the entire name when the sound cue and “speak” legend appeared. Subjects had 4 s to provide an answer. On this day, the first syllable of the names was not presented. However, participants did receive feedback on each trial. The inter-item duration was the same as on Day 1.

##### Free recall (item memory - names)

Subjects were instructed to recall aloud all the names that they could remember from the experiment for 1 min.

##### Recognition (item memory - faces)

The ten previously learned faces were presented randomly, along with ten new faces on screen. During the test, faces appeared one at a time, and participants were instructed to make an OLD/NEW judgment aloud. All responses were recorded.

##### Additional measures

Before finishing, participants were asked face-to-face and aloud: (1) how many individual faces they had seen during the entire face-name task at testing (open question); (2) the number of repetitions of each individual face at testing (open question); (3) overall confidence in their responses at testing considering a 1–10 subjective scale.

### Analytic strategy

Data analysis was conducted using R 4.0.5 within the Bayesian framework. Bayesian posterior estimation provides many advantages, such as robust parameter estimation, their uncertainty, and the ability to quantify evidence for or against models [40]. Mixed-effects logistic regression models were implemented using the *brms* package on each memory evaluation. Associative memory accuracy (face-name pairs) was modeled as a function of group (young adults, older adults, and aMCI patients), reactivation type (reactivation vs reactivation control) and Block (fixed effects). For item memory, group, and reactivation were used as fixed effects with the inclusion of Stimulus type (old/ new) in memory recognition analysis. In all analyzes, subject-level and item-level (face identity) intercepts were used as random effects, and the reactivation control served as reference. Weakly informative Cauchy priors with location parameters of 0 and scale parameters of 2.5 (for the fixed effects) and ten (for the random effects) were specified. Additionally, we conducted a prior sensitivity analysis to test the influence of priors on the posterior distribution. Overall, priors had a neglectable influence on the reported results (Supplementary Materials S5). All models indicated convergence, according to the Gelman–Rubin r̂ statistic (*r̂* < 1.01), and were fitted using four chains with 5000 iterations and 2000 warm-up iterations. For each analysis, we implemented different models that varied in complexity and number of fixed effects to evaluate their importance and their interactions. Model comparison was based on Pareto Smoothed importance sampling Leave-One-Out Cross-Validation (LOO-CV) which computes the difference in Expected Log Pointwise Predictive Density (ELPD Difference). ELPD difference quantifies the predictive accuracy of the best-performing model relative to the others [41]. Hence, models that underperform the winning model, are expected to have negative ELPD differences. We also calculated the Bayes Factor (BF) using bridge sampling for comparing models that included or not different fixed effects. BF was based on the ratio of evidence comparing one alternative model against the null model (BF_10_). A BF <1 indicates that both models are equally likely, a BF >3 could be interpreted as moderate evidence, and a BF >10 provides strong evidence in favor of the model [42]. *Tidybayes* and *emmeans* R packages were used to generate samples for each marginal mean and to create contrasts between conditions of interest (main effects and interactions). All analyses used the mean as the posterior point estimate and the 95% Highest Density Interval (HDI) as a measure of uncertainty. The HDI conveys the most probable values in the posterior. Parameter estimates can be thought as statistically meaningful (akin to statistical significance in the frequentist approach) if their HDI excluded zero. Additional measures (number of faces, repetitions, and overall response confidence), were analyzed by means of independent Bayesian ANOVA implemented in *JASP* with default priors [43]. Finally, group comparisons between older adults and aMCI patients in demographic variables and the neuropsychological battery, were performed in *JASP* using Bayesian independent *t*-test with default priors (Supplementary Materials S1).

## Results

### Associative memory (face-name pairs) acquisition and evaluation

**Fig. 2:**
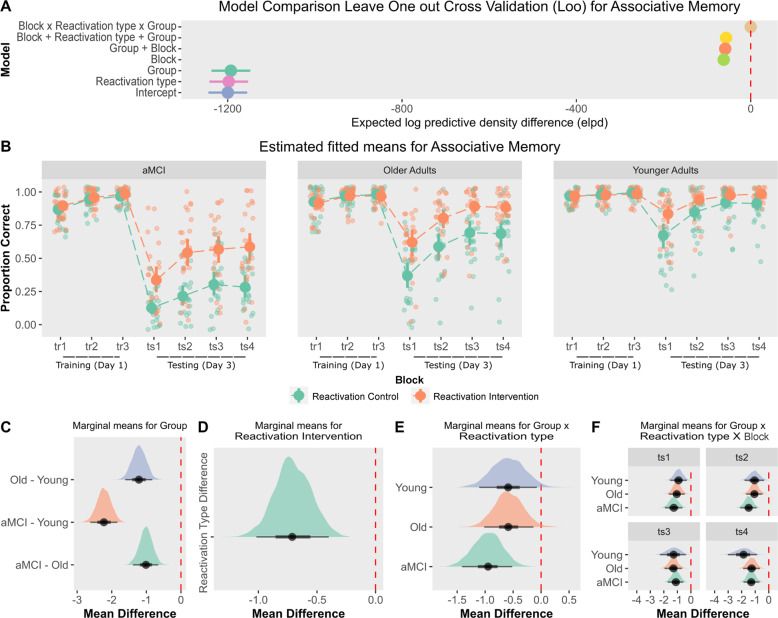
Associative memory performance (face-name pairs). **A** Model comparison: leave-one-out cross-validation indicated that the interaction model had the highest prediction accuracy (ELPD difference). **B** Estimated fitted means with 95% HDI (Accuracy) across training (tr1–tr3) and testing (ts1–ts4) blocks. Small points show raw accuracy. Groups that received the reactivation intervention showed improved memory retention on Day 3. **C**–**F** Posterior marginal means with 95% HDI and contrasts, showed that the reactivation intervention improved memory retention across groups and testing blocks.

Model comparison showed that the inclusion of block × group (younger adults, older adults and aMCI patients) × reactivation type (reactivation intervention vs reactivation control) interaction had the highest prediction accuracy (ELPD difference) relative to the other less complex models (Fig. 2A; for models posterior estimates and a full model comparison of LOO-CV see Supplementary Materials S2). Similarly, a Bayesian modeling averaging analysis [44] provided additional support for the interaction inclusion and all the other predictors as well (each BF inclusion > 3000).

As depicted in Fig. 2B, on Day 1 memory accuracy in the face-name training was similar across groups. In the last training block, all participants responded correctly to at least 60% of the names and there were no differences between groups (group × block contrast, aMCI vs young adults (tr3) *M*_diff_ = −0.89 [−1.90, 0.13], BF = 0.3; aMCI vs older adults (tr3) *M*_diff_ = 0.13 [−0.64, 0.99], BF = 0.04; older Aadults vs young adults (tr3), *M*_diff_ = −1.02 [−1.92, 0.09], BF = 0.19) or reactivation Type (reactivation type × block contrast *M*_diff_ = 0.18 [−0.61, 0.95], BF = 0.04). We observed a considerable reduction in memory performance at testing (Day 3). As expected, younger adults have the highest overall memory retention (Fig. 2C, group main effect BF > 1000, aMCI vs younger adults *M*_diff_ = −2.23 [−2.62, −1.84], BF > 1000; older adults vs younger adults *M*_diff_ = −1.21 [−1.61, −0.84], BF > 1000) and older adults performed better than aMCI patients (aMCI vs older adults *M*_diff_ = −1.01 [−1.38, −0.65], BF = 499.4). Notably, memory accuracy was drastically different across groups depending on the reactivation type (Fig. 2D, reactivation main effect BF > 1000, *M*_dif_ = −0.71 [−1.01, −0.40], BF = 267.4). From the first testing block (ts1) to the last one (ts4), we found evidence that groups that received the reactivation intervention had a better performance than those which received the reactivation control (Fig. 2E, group × reactivation type interaction main effect BF > 1000, aMCI *M*_diff_ = −0.95 [−1.46, −0.47], BF = 0.53; older adults = *M*_diff_ = −0.62 [−1.06, −0.08], BF = 0.283; *M*_diff_ = −0.59 [−1.15, −0.06], BF = 0.193). A trial by trial analysis provided more evidence of a strengthening effect on episodic memory retention produced by the reactivation intervention (Fig. 2F, group × block × reactivation type interaction main effect BF > 1000, aMCI patients (ts1) *M*_diff_ = −1.26 [−1.92, −0.63], BF = 103.06; older adults (ts1) *M*_diff_ = −1.04 [−1.60, −0.45], BF = 4.27; younger adults (ts1) *M*_diff_ = −0.91 [−1.55, −0.33], BF = 3.13). Interestingly, the reactivation intervention had the strongest effect in aMCI patients on memory retention (27% memory improvement relative to the reactivation control, Cohen’s *d* = 1.14 [0.53, 1.73]), followed by older adults (20%, Cohen’s *d* = 0.94 [0.36, 1.4]) and younger adults (11%, Cohen’s *d* = 0.66 [0.09, 1.23]).

### Item memory evaluation

#### Names free recall

**Fig. 3:**
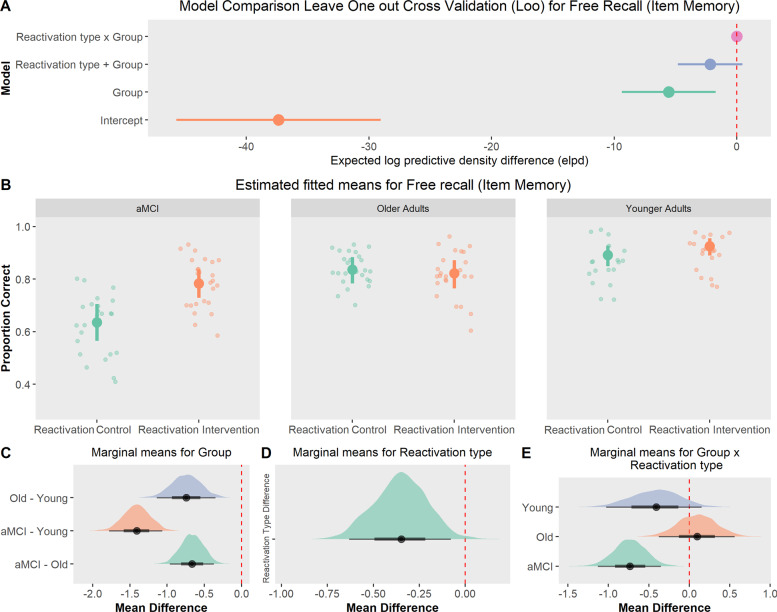
Free recall (item memory). **A** Model Comparison: Leave-one-out cross-validation indicated that the interaction model or main effects model had the highest prediction accuracy (ELPD difference). **B** Estimated fitted means with 95% HDI (Recall) across groups and reactivation type. Small points show raw recall. Groups that received the reactivation intervention showed a slightly better memory retention on Day 3. **C**–**E** Posterior marginal means with 95% HDI and contrasts, showed that the reactivation intervention improved item memory only in aMCI patients.

LOO-CV analysis indicated that the inclusion of both group and reactivation type as predictors improved the prediction accuracy of the models (Fig. 3A; see full model comparison in Supplementary Materials S3). Evidence for an interaction between group and reactivation type was inconclusive (ELPD difference between models with and without interaction term = −2.17, SE = 2.6; BF_inclusion_ = 1.26). Model averaging also supported the inclusion of both main effects (group BF_inclusion_ > 10000 and reactivation type BF_inclusion_ = 6.2). Overall, younger adults recalled more names than older adults (Fig. 3B and C, *M*_diff_ = −0.74 [−1.14, −0.35], BF = 9.34) and aMCI patients (*M*_diff_ = −1.41 [−1.76, −1.06], BF > 1000). Evidence for a reactivation type effect was modest (Fig. 3D, *M*_diff_ = −0.35 [−0.63, −0.08], BF = 1.60). Interestingly, contrast analysis revealed that aMCI patients in the reactivation intervention had better Item memory retention than the reactivation control (Fig. 3E, *M*_diff_ = −0.75 [−1.13, −0.36], BF = 22.10). For young adults and older adults we found no support for any difference between conditions (*M*_diff_ = −0.37 [−1.03, 0.15], BF = 0.11 and *M*_diff_ = 0.12 [−0.39, 0.55], BF = 0.04, respectively).

#### Face recognition

**Fig. 4:**
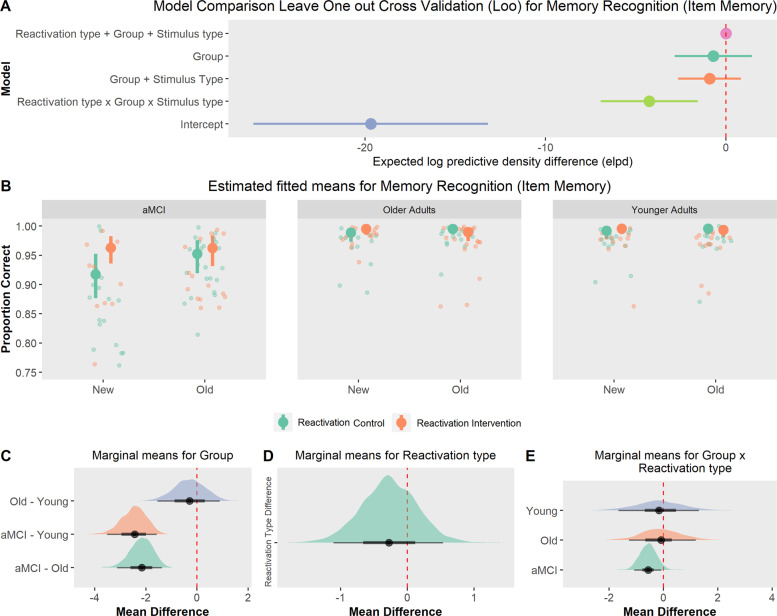
Memory recognition (item memory). **A** Model comparison: leave-one-out cross-validation suggested that the inclusion of group as predictor improved model performance with no clear benefit of the other predictors. **B** Estimated fitted means with 95% HDI (memory recognition) across groups, reactivation type and stimulus type (old/new). Small points show raw responses. All groups had a robust performance (>90% of correct responses). **C**–**E** Posterior marginal means with 95% HDI and contrasts, showed that the reactivation intervention improved memory recognition only in aMCI patients.

Memory recognition was near ceiling across groups and conditions (between 90% and 99% correct responses). Main effects only and interaction models showed better prediction accuracy than the null model (Fig. 4A). However, model comparison did not favor any specific model (see full model comparison in Supplementary Materials S4; also all BF_inclusion_ < 1). aMCI patients had lower memory recognition than older adults (Fig. 4B and C, *M*_diff_ = −2.16 [−3.03, −1.30], BF > 1000) and younger adults (*M*_diff_ = −2.44 [−3.45, −1.52], BF > 1000). We found no evidence for a reactivation type effect (Fig. 4D, *M*_diff_ = −0.27 [−1.13, 0.50], BF = 0.06). Thus, contrast analysis suggested an anecdotal evidence for a reactivation effect in aMCI patients (Fig. 4E, *M*_diff_ = −0.56 [−1.17, −0.02], BF = 0.6) but any evidence in older adults (*M*_diff_ = −0.11 [−1.57, 1.45], BF = 0.07) or younger adults (*M*_diff_ = 0.17 [−2.07, 1.52], BF = 0.09).

### Additional measures

The number of perceived faces and their repetitions after testing (Day 3) was similar across groups and conditions (Supplementary Materials S6). A Bayesian ANOVA group × reactivation type for perceived faces and number of repetitions performed in *JASP*, did not support strong evidence for a model with group or reactivation type as predictors (all BF´s model relative to the null model and BF_inclusion_ effect < 3). Conversely, overall participants’ confidence was different based on group and reactivation type. A Bayesian ANOVA favored the main effects and the inclusion of the interaction relative to the null model (group × reactivation type model, BF > 10000 and BF_inclusion_ = 7.81). Independent Bayesian *T*-test´s revealed that aMCI patients in the reactivation intervention (*M* = 9.12 [8.58, 9.65]) had higher levels of response confidence relative to the reactivation control (*M* = 8.36 [7.70, 9.01]; BF = 51.91). However, this effect was absent in older adults and younger adults (BF < 1).

## Discussion

The study of memory reactivation and stabilization at long delays in populations with memory deficits is absent. Only recent work examined memory reactivation in older adults with limited evidence [31, 32, 34]. The current findings provide evidence that memory reactivation facilitates episodic memory stabilization and improves memory retention at long delays across the lifespan. More importantly, we showed that a delayed reactivation intervention strengthened memory performance in a population with objective episodic memory deficits (aMCI patients), in both associative and item memory, suggesting that those with the weakest memory ability benefited more from the intervention. Finally, this proof-of-concept study supports the feasibility of memory reactivation-based interventions in clinical settings, such as cognitive stimulation or rehabilitation of memory deficits.

These results are aligned with studies of targeted memory reactivation during sleep and post-encoding awake reactivation, which showed memory reactivation as a stabilization mechanism [19, 24, 45, 46]. Several results established the memory benefits of quiet rest and sleep shortly after learning, even in amnesic patients [15, 47]. Initially acquired memories are thought to be maturated by post-encoding processes during offline or “quiet restful” periods [17, 25, 48]. Hippocampal neurons during slow-wave sleep fire in fast oscillations (sharp-wave ripples) co-occurring with rhythmic thalamocortical activity (spindles). Neuroimaging studies in humans also found evidence of post-encoding reactivation in the similarity of hippocampal activity between encoding and post-encoding patterns [17, 24]. Notably, the magnitude of this reactivation predicts subsequent memory [24, 49]. Thus, memory reactivation enables the stabilization of recently acquired memories, and in consequence, their maintenance and protection from interference [20, 21]. Given the well-known function of the hippocampus in episodic memory formation, memory reactivation, and its atrophy in MCI patients, we assume that the hippocampal formation may be critically recruited during the reactivation intervention in order to promote memory stabilization and strengthening. However, future research should use neuroimaging techniques and target hippocampal-cortical interactions and their contribution in episodic memory strengthening.

Previous work on memory reconsolidation in animals and humans, also demonstrated that re-exposure to learned cues after memory consolidation strengthens memory retention, prevents forgetting, and improves memory persistence [27–29]. However, not all reminders are equally effective at stabilizing memory or triggering the reconsolidation process. Cues that involve a discrepancy between what is expected and what actually occurs (Prediction Error), are proposed to drive memory reactivation-reconsolidation [26]. A recent work by Forcato and colleagues [38] provided evidence that only reminders that included a prediction error (incomplete reminders) stabilized memory in the long term. We believe that our reactivation-based intervention followed similar principles in the “reminder” construction and thus promoted memory strengthening. Besides its critical role in strengthening specific memories, memory reactivation may promote memory integration into cortical circuits [24, 50]. Memories are thought to be gradually transformed and stored in interconnected networks by system-level consolidation [51]. This long-range process allows the extraction of regularities across experiences and its generalization across memories. In this sense, the integration of new memories into previous knowledge increases the efficiency of the memory network. For example, Bavassi et al. [52] found that memories strengthened by the reactivation-reconsolidation process presented a more interconnected network between brain regions (denser network with increased values of clustering coefficient) relative to retrained memories.

This study has several limitations. It was designed to test changes in episodic memory retention as a product of a specific reactivation intervention. Although the results supported this idea, the evidence provided for its effect is relative rather than absolute. That is, we demonstrate the specificity and efficacy of a reactivation intervention with respect to a reactivation control but in the absence of non-reactivated groups. Non-reactivated groups would have shown a baseline to assess the absolute effects of the reactivation intervention and assessed whereas the reactivation control improved or impaired memory retention. Although, previous studies that have used similar reactivations to the reactivation control, indicate that this procedure does not alter long-term memory retention, as the degree of prediction error would be insufficient with respect to the target reactivation [26–28, 53, 54]. Another limitation of this work resides in the evaluation of item memory. The fact that memory was evaluated after associative memory could have biased the results and generated a ceiling effect. Future studies should contemplate more directed experimental designs to examine how memory reactivation affects item memory retention.

This is the first study to demonstrate a specific improvement in episodic memory retention in a population with objective memory deficit (aMCI) using a reactivation intervention. Moreover, this benefit was found in an associative task which resembles common memory difficulties in the elderly. In the last decade, a large body of studies proposed the therapeutic utility of memory reactivation-reconsolidation to modify dysfunctional memories (i.e., phobias, traumas, etc; [55]) aimed to develop “technologies of forgetting”. Conversely, here we propose a reactivation intervention to produce enduring changes in memory stability and retention, aiming to develop more efficient “technologies of remembering”. Current cognitive stimulation/rehabilitation treatments for memory deficits emphasize acquisition and retrieval processes in order to strengthen specific and relevant memories such as caregiver names, addresses, or phones [8]. However, as amnesic patients commonly have consolidation impairments, this information is fragile and rapidly susceptible to disruption [12, 13, 16]. Hence, targeting memory stabilization in the time interval between memory acquisition and later retrieval, could be helpful to improve memory retention and to increase its later probability of retrieval. In this sense, reactivation-based interventions could be easily applied in the context of cognitive stimulation/rehabilitation and contribute to compensate memory deficits in clinical settings.

## Supplementary information

Supplemental Material

## Data Availability

Raw data used in the study can be found here: https://osf.io/r7qbn/.

## Code availability

Code for performing the Bayesian analyses in R can be provided upon request.
