# Supplementary material for: Improvement of episodic memory retention by a memory reactivation intervention across the lifespan: from younger adults to amnesic patients
Source: Transl Psychiatry. 2022 Apr 5;12:144. doi: 10.1038/s41398-022-01915-z (PMC8983690; doi:10.1038/s41398-022-01915-z)
Supplement: Supplementary file 1 — Supplemental Material [file 41398_2022_1915_MOESM1_ESM.docx]

**Improvement of episodic memory retention by a memory reactivation intervention across the lifespan: from younger adults to amnesic patients.**

Rodrigo S. Fernández^1-2*^, Soledad Picco^1-2^, Juan Cruz Beron^1-2^, Luz Bavassi^1-2^, Jorge Campos^3^, Ricardo F. Allegri^3^, María E. Pedreira^1-2*^.

1- Instituto de Fisiología, Biología Molecular y Neurociencias (IFIBYNE - CONICET), Ciudad de Buenos Aires, Argentina.

2- Facultad de Ciencias Exactas y Naturales Universidad de Buenos Aires, Ciudad de Buenos Aires, Argentina.

3- Department of Cognitive Neurology, Neuropsychiatry and Neuropsychology, Fleni, Buenos Aires, Argentina.

**Sections:**

S1- Baseline characteristics and neuropsychological assessment of Older adults and aMCI patients.

S2- Associative Memory (face-name pairs) posterior models estimates and full LOO-CV analysis.

S3- Item Memory (Free Recall) posterior models estimates and full LOO-CV analysis.

S4- Item Memory (Recognition) posterior models estimates and full LOO-CV analysis.

S5- Prior sensitivity analysis.

1. **Baseline characteristics and neuropsychological assessment of Older adults and aMCI patients.**

|  |  |  | **95 % CI** |  |  |
| --- | --- | --- | --- | --- | --- |
|  | **Group** | **Mean** | **Lower** | **Upper** | **BF₁₀ (t-test)** |
| **Age** | Older Adults | 73.84 | 72.33 | 75.34 | 0.34 |
|  | aMCI | 72.42 | 70.19 | 74.64 |  |
| **Gender** | Older Adults | 0.46 | 0.31 | 0.6 | 0.211 |
|  | aMCI | 0.46 | 0.31 | 0.6 |  |
| **Years of Education** | Older Adults | 13.24 | 12.65 | 13.82 | 0.388 |
|  | aMCI | 13.78 | 13.06 | 14.49 |  |
| **MMSE** | Older Adults | 29.22 | 28.94 | 29.49 | > 1000 |
|  | aMCI | 26.84 | 26.44 | 27.23 |  |
| **Logical Memory Test (immediate)** | Older Adults | 25.5 | 23.92 | 27.07 | > 1000 |
|  | aMCI | 13.58 | 12.68 | 14.47 |  |
| **Logical Memory Test (delayed)** | Older Adults | 20.34 | 18.5 | 22.17 | > 1000 |
|  | aMCI | 8.44 | 7.25 | 9.62 |  |
| **RAVLT (immediate)** | Older Adults | 40.7 | 38.1 | 43.29 | > 1000 |
|  | aMCI | 26.28 | 24.7 | 27.85 |  |
| **RAVLT (delayed)** | Older Adults | 6.62 | 5.98 | 7.25 | > 1000 |
|  | aMCI | 2.94 | 2.26 | 3.61 |  |
| **Rey-Osterrieth Complex Figure (copy)** | Older Adults | 35.02 | 33.97 | 36.06 | 1.085 |
|  | aMCI | 32.78 | 30.69 | 34.86 |  |
| **Rey-Osterrieth Complex Figure (recall)** | Older Adults | 15.58 | 13.97 | 17.18 | 591.289 |
|  | aMCI | 11.1 | 9.79 | 12.41 |  |
| **Boston Naming Test** | Older Adults | 26.44 | 25.03 | 27.84 | 0.222 |
|  | aMCI | 26.72 | 25.82 | 27.61 |  |
| **Semantic Fluency** | Older Adults | 16.62 | 15.09 | 19.14 | 1.2 |
|  | aMCI | 15.78 | 14.6 | 16.95 |  |
| **Phonological Fluency** | Older Adults | 14.82 | 13.71 | 15.9 | 0.382 |
|  | aMCI | 13.8 | 12.42 | 15.17 |  |
| **Digit Span Forward** | Older Adults | 6.4 | 6.07 | 6.73 | 0.992 |
|  | aMCI | 5.98 | 5.67 | 6.28 |  |
| **Digit Span Backward** | Older Adults | 4.88 | 4.55 | 5.2 | 56.94 |
|  | aMCI | 4.02 | 3.67 | 4.36 |  |
| **Digit Symbol-Coding** | Older Adults | 45.04 | 41.43 | 48.64 | 13.253 |
|  | aMCI | 38.02 | 35.23 | 40.8 |  |
| **TMTa** | Older Adults | 37.02 | 34.42 | 39.62 | > 1000 |
|  | aMCI | 49.56 | 45.04 | 54.07 |  |
| **TMTb** | Older Adults | 87.08 | 80.46 | 93.69 | > 1000 |
|  | aMCI | 124.56 | 118.8 | 130.31 |  |

**Mean estimates by Block, Reactivation type and Group of the Associative memory evaluation (Day 3)**

| **Block** | **Reactivation Type** | **Group** | **Proportion Correct** | **SD** |
| --- | --- | --- | --- | --- |
| **tr1** | *Reactivation Intervention* | Old | 0.90 | 0.30 |
| **tr1** | *Reactivation Intervention* | aMCI | 0.87 | 0.33 |
| **tr1** | *Reactivation Intervention* | Young | 0.96 | 0.20 |
| **tr1** | *Reactivation Control* | Old | 0.91 | 0.29 |
| **tr1** | *Reactivation Control* | aMCI | 0.87 | 0.34 |
| **tr1** | *Reactivation Control* | Young | 0.95 | 0.21 |
| **tr2** | *Reactivation Intervention* | Old | 0.96 | 0.19 |
| **tr2** | *Reactivation Intervention* | aMCI | 0.95 | 0.22 |
| **tr2** | *Reactivation Intervention* | Young | 0.97 | 0.18 |
| **tr2** | *Reactivation Control* | Old | 0.96 | 0.20 |
| **tr2** | *Reactivation Control* | aMCI | 0.94 | 0.25 |
| **tr2** | *Reactivation Control* | Young | 0.97 | 0.17 |
| **tr3** | *Reactivation Intervention* | Old | 0.96 | 0.20 |
| **tr3** | *Reactivation Intervention* | aMCI | 0.98 | 0.13 |
| **tr3** | *Reactivation Intervention* | Young | 0.98 | 0.14 |
| **tr3** | *Reactivation Control* | Old | 0.98 | 0.15 |
| **tr3** | *Reactivation Control* | aMCI | 0.96 | 0.20 |
| **tr3** | *Reactivation Control* | Young | 0.99 | 0.09 |
| **ts1** | *Reactivation Intervention* | Old | 0.61 | 0.49 |
| **ts1** | *Reactivation Intervention* | aMCI | 0.36 | 0.48 |
| **ts1** | *Reactivation Intervention* | Young | 0.80 | 0.40 |
| **ts1** | *Reactivation Control* | Old | 0.38 | 0.49 |
| **ts1** | *Reactivation Control* | aMCI | 0.13 | 0.34 |
| **ts1** | *Reactivation Control* | Young | 0.66 | 0.47 |
| **ts2** | *Reactivation Intervention* | Old | 0.78 | 0.42 |
| **ts2** | *Reactivation Intervention* | aMCI | 0.54 | 0.50 |
| **ts2** | *Reactivation Intervention* | Young | 0.92 | 0.27 |
| **ts2** | *Reactivation Control* | Old | 0.58 | 0.49 |
| **ts2** | *Reactivation Control* | aMCI | 0.22 | 0.42 |
| **ts2** | *Reactivation Control* | Young | 0.82 | 0.39 |
| **ts3** | *Reactivation Intervention* | Old | 0.87 | 0.33 |
| **ts3** | *Reactivation Intervention* | aMCI | 0.56 | 0.50 |
| **ts3** | *Reactivation Intervention* | Young | 0.97 | 0.18 |
| **ts3** | *Reactivation Control* | Old | 0.68 | 0.47 |
| **ts3** | *Reactivation Control* | aMCI | 0.31 | 0.46 |
| **ts3** | *Reactivation Control* | Young | 0.90 | 0.30 |
| **ts4** | *Reactivation Intervention* | Old | 0.86 | 0.34 |
| **ts4** | *Reactivation Intervention* | aMCI | 0.58 | 0.50 |
| **ts4** | *Reactivation Intervention* | Young | 0.98 | 0.14 |
| **ts4** | *Reactivation Control* | Old | 0.67 | 0.47 |
| **ts4** | *Reactivation Control* | aMCI | 0.29 | 0.45 |
| **ts4** | *Reactivation Control* | Young | 0.89 | 0.32 |

1. **Associative Memory (face-name pairs) posterior models estimates and full LOO-CV analysis.**

Results of generalized linear mixed effects model for memory accuracy (note: coefficients on the log-odds scale). Posterior mean Estimate, Maximum a Posteriori Probability (MAP),95% highest density interval (HDI), Bayes Factor (BF) and Rhat diagnostic**.**

| 1. **Null model**: Accuracy ~ 1 + (1\|Id) | | | | | | |
| --- | --- | --- | --- | --- | --- | --- |
|  | | | **95% HDI** | |  | |
| **Parameter** | **Mean** | **MAP** | **Lower** | **Upper** | **BF** | **Rhat** |
| Intercept | 1.53 | 1.51 | 1.34 | 1.73 | 5.02E+16 | 1.01 |
| Subject random effect (Intercept) | 1.14 | 1.14 | 1 | 1.31 | 1.19E+19 | 1 |

1. **Interaction Model (Reported):** Accuracy ~ Group * Reactivation type*Block + (1|Id) + (1|Stim)

|  | | | **95% HDI** | |  | |
| --- | --- | --- | --- | --- | --- | --- |
| **Parameter** | **Mean** | **MAP** | **Lower** | **Upper** | **BF** | **Rhat** |
| Intercept | 1.91 | 1.87 | 1.43 | 2.38 | 3.42E+05 | 1 |
| **Group: Main Effect** | | | | | | |
| Group Older Adults | 0.67 | 0.68 | 0 | 1.3 | 8.10E-01 | 1 |
| Group Younger Adults | 1.59 | 1.51 | 0.76 | 2.33 | 2.43E+02 | 1 |
| **Reactivation type: Main Effect** | | | | | | |
| Reactivation type | 0.28 | 0.3 | -0.31 | 0.93 | 1.60E-01 | 1 |
| **Block: Main Effect** | | | | | | |
| Block Tr2 | 0.95 | 0.94 | 0.39 | 1.51 | 1.43E+01 | 1 |
| Block Tr3 | 1.55 | 1.39 | 0.91 | 2.29 | 1.79E+02 | 1 |
| Block Ts1 | -3.86 | -3.86 | -4.35 | -3.35 | 1.34E+22 | 1 |
| Block Ts2 | -3.22 | -3.15 | -3.69 | -2.78 | 8.90E+10 | 1 |
| Block Ts3 | -2.75 | -2.73 | -3.18 | -2.31 | 1.39E+13 | 1 |
| Block Ts4 | -2.85 | -2.84 | -3.27 | -2.39 | 3.27E+12 | 1 |
| **Group x Reactivation type: Interaction** | | | | | | |
| Group Older Adults x Reminder | -0.42 | -0.43 | -1.33 | 0.49 | 2.10E-01 | 1 |
| Group Younger Adults x Reminder | -0.26 | -0.38 | -1.26 | 0.81 | 2.00E-01 | 1 |
| **Group x Block: Interaction** | | | | | | |
| Group Older Adults x Block Tr2 | -0.01 | 0 | -0.95 | 0.85 | 1.30E-01 | 1 |
| Group Younger Adults x Block Tr2 | -0.46 | -0.35 | -1.52 | 0.56 | 2.60E-01 | 1 |
| Group Older Adults x Block Tr3 | -0.08 | -0.14 | -1.14 | 1.02 | 1.80E-01 | 1 |
| Group Younger Adults x Block Tr3 | 0.19 | 0.23 | -1.39 | 1.62 | 2.40E-01 | 1 |
| Group Older Adults x Block Ts1 | 0.74 | 0.83 | 0.05 | 1.41 | 1.41E+00 | 1 |
| Group Younger Adults x Block Ts1 | 1.09 | 1.04 | 0.33 | 1.85 | 6.01E+00 | 1 |
| Group Older Adults x Block Ts2 | 1.01 | 1.07 | 0.39 | 1.69 | 8.36E+00 | 1 |
| Group Younger Adults x Block ts2 | 1.46 | 1.51 | 0.67 | 2.21 | 3.91E+01 | 1 |
| Group Older Adults x Block Ts3 | 1 | 0.92 | 0.39 | 1.65 | 1.04E+01 | 1 |
| Group Younger Adults x Block Ts3 | 1.72 | 1.79 | 0.92 | 2.54 | 1.15E+02 | 1 |
| Group Older Adults x Block Ts4 | 1.06 | 1.14 | 0.41 | 1.68 | 1.21E+01 | 1 |
| Group Younger Adults x Block Ts4 | 1.73 | 1.82 | 0.91 | 2.51 | 1.31E+02 | 1 |
| **Reactivation type x Block: Interaction** | | | | | | |
| Reactivation type x Block Tr2 | 0.06 | 0.08 | -0.72 | 0.92 | 1.20E-01 | 1 |
| Reactivation type x Block Tr3 | 0.59 | 0.67 | -0.56 | 1.71 | 2.70E-01 | 1 |
| Reactivation type x Block Ts1 | 0.98 | 1.01 | 0.38 | 1.66 | 1.02E+01 | 1 |
| Reactivation type x Block Ts2 | 1.21 | 1.14 | 0.63 | 1.81 | 2.19E+02 | 1 |
| Reactivation type x Block Ts3 | 0.84 | 0.77 | 0.22 | 1.42 | 4.77E+00 | 1 |
| Reactivation type x Block Ts4 | 1.01 | 1.03 | 0.42 | 1.64 | 4.75E+01 | 1 |
| **Group x Reactivation type x Block: Interaction** | | | | | | |
| Group Older Adults x Reactivation type x Block Tr2 | 0.15 | 0.16 | -1.12 | 1.47 | 2.10E-01 | 1 |
| Group Younger Adults x Reactivation type x Block Tr2 | -0.27 | -0.18 | -1.64 | 1.14 | 2.60E-01 | 1 |
| Group Older Adults x Reactivation type x Block Tr3 | -1.01 | -0.93 | -2.57 | 0.54 | Inf | 1 |
| Group Younger Adults x Reactivation type x Block Tr3 | -1.48 | -1.47 | -3.45 | 0.6 | 9.90E-01 | 1 |
| Group Older Adults x Reactivation type x Block Ts1 | 0.19 | 0.2 | -0.75 | 1.04 | 1.60E-01 | 1 |
| Group Younger Adults x Reactivation type x Block Ts1 | -0.08 | -0.11 | -1.13 | 0.95 | 1.80E-01 | 1 |
| Group Older Adults x Reactivation type x Block Ts2 | -0.01 | -0.03 | -0.91 | 0.77 | 1.40E-01 | 1 |
| Group Younger Adults x Reactivation type x Block Ts2 | -0.16 | -0.16 | -1.2 | 0.94 | 1.40E-01 | 1 |
| Group Older Adults x Reactivation type x Block Ts3 | 0.59 | 0.68 | -0.31 | 1.47 | 3.80E-01 | 1 |
| Group Younger Adults x Reactivation type x Block Ts3 | 0.44 | 0.48 | -0.8 | 1.61 | 2.40E-01 | 1 |
| Group Older Adults x Reactivation type x Block Ts4 | 0.39 | 0.44 | -0.5 | 1.25 | 1.90E-01 | 1 |
| Group Younger Adults x Reactivation type x Block Ts4 | 0.86 | 0.67 | -0.45 | 2.18 | 4.70E-01 | 1 |
| **Group-Level Effects** | | | | | | |
| Subject random effect (Intercept) | 0.8 | 0.79 | 0.69 | 0.93 | 2.99E+13 | 1 |
| Stimulus random effect (Intercept) | 0.14 | 0.12 | 0.04 | 0.28 | 5.70E-01 | 1 |

1. **Main effects Model:** Accuracy ~ Group + Reactivation type + Block + (1|Id) + (1|Stim)

|  | | | **95% HDI** | |  | |
| --- | --- | --- | --- | --- | --- | --- |
| **Parameter** | **Mean** | **MAP** | **Lower** | **Upper** | **BF** | **Rhat** |
| Intercept | 1.22 | 1.23 | 0.87 | 1.56 | 1.52E+05 | 1 |
| **Group: Main Effect** | | | | | | |
| Group Older Adults | 1.3 | 1.29 | 0.94 | 1.63 | 5.41E+04 | 1 |
| Group Younger Adults | 2.61 | 2.57 | 2.24 | 2.95 | 3.43E+18 | 1 |
| **Reactivation type: Main Effect** | | | | | | |
| Reactivation type | 0.96 | 0.96 | 0.68 | 1.24 | 6.13E+05 | 1 |
| **Block: Main Effect** | | | | | | |
| Block Tr2 | 0.9 | 0.88 | 0.57 | 1.22 | 2.01E+04 | 1 |
| Block Tr3 | 1.48 | 1.48 | 1.11 | 1.88 | 5.53E+05 | 1 |
| Block Ts1 | -3.04 | -3.05 | -3.28 | -2.81 | 2.33E+23 | 1 |
| Block Ts2 | -2.16 | -2.15 | -2.39 | -1.92 | 4.05E+13 | 1 |
| Block Ts3 | -1.72 | -1.72 | -1.95 | -1.5 | 3.21E+13 | 1 |
| Block Ts4 | -1.74 | -1.73 | -1.97 | -1.5 | 1.21E+12 | 1 |
| **Group-Level Effects** | | | | | | |
| Subject random effect (Intercept) | 0.78 | 0.78 | 0.67 | 0.91 | 9.16E+10 | 1 |
| Stimulus random effect (Intercept) | 0.14 | 0.12 | 0.03 | 0.25 | 7.80E-01 | 1 |
|  |  |  |  |  |  |  |

1. **Condition + Block Model:** Accuracy ~ Group + Block + (1|Id) + (1|Stim)

|  | | | **95% HDI** | |  | |
| --- | --- | --- | --- | --- | --- | --- |
| **Parameter** | **Mean** | **MAP** | **Lower** | **Upper** | **BF** | **Rhat** |
| Intercept | 1.7 | 1.7 | 1.34 | 1.99 | 1.26E+09 | 1 |
| **Group: Main Effect** | | | | | | |
| Group Older Adults | 1.29 | 1.27 | 0.89 | 1.69 | 2.33E+03 | 1 |
| Group Younger Adults | 2.62 | 2.61 | 2.19 | 3.02 | 3.00E+14 | 1.01 |
| **Block: Main Effect** | | | | | | |
| Block Tr2 | 0.91 | 0.91 | 0.58 | 1.25 | 4.33E+02 | 1 |
| Block Tr3 | 1.48 | 1.46 | 1.1 | 1.89 | 1.54E+04 | 1 |
| Block Ts1 | -3.04 | -3.03 | -3.26 | -2.8 | 2.02E+25 | 1 |
| Block Ts2 | -2.15 | -2.14 | -2.36 | -1.91 | 1.09E+19 | 1 |
| Block Ts3 | -1.72 | -1.7 | -1.94 | -1.49 | 1.78E+13 | 1 |
| Block Ts4 | -1.73 | -1.74 | -1.96 | -1.5 | 3.49E+14 | 1 |
| **Group-Level Effects** | | | | | | |
| Subject random effect (Intercept) | 0.93 | 0.93 | 0.8 | 1.07 | 1.43E+18 | 1 |
| Stimulus random effect (Intercept) | 0.14 | 0.12 | 0.03 | 0.26 | 5.30E-01 | 1 |

1. **Group Model:** Accuracy ~ Group + (1|Id) + (1|Stim)

|  | | | **95% HDI** | | |  | | |  |
| --- | --- | --- | --- | --- | --- | --- | --- | --- | --- |
| **Parameter** | **Mean** | **MAP** | | **Lower** | **Upper** | | **BF** | **Rhat** | |
| Intercept | 0.51 | 0.51 | | 0.28 | 0.73 | | 8.73E+00 | 1 | |
| **Group: Main Effect** | | | | | | | | |  |
| Group Older Adults | 0.96 | 0.95 | | 0.67 | 1.27 | | 8.84E+03 | 1 | |
| Group Younger Adults | 2.07 | 2.08 | | 1.72 | 2.39 | | 2.46E+10 | 1 | |
| **Group-Level Effects** | | | | | | | | |  |
| Subject random effect (Intercept) | 0.72 | 0.72 | | 0.61 | 0.83 | | 3.19E+10 | 1 | |
| Stimulus random effect (Intercept) | 0.09 | 0.08 | | 0 | 0.17 | | 2.80E-01 | 1 | |

1. **Reactivation type model:** Accuracy ~ Reactivation type + (1|Id) + (1|Stim)

|  | | | **95% HDI** | |  | |
| --- | --- | --- | --- | --- | --- | --- |
| **Parameter** | **Mean** | **MAP** | **Lower** | **Upper** | **BF** | **Rhat** |
| Intercept | 1.15 | 1.16 | 0.89 | 1.42 | 200 | 1.01 |
| **Reactivation type: Main Effect** | | | | | | |
| Reactivation type | 0.75 | 0.78 | 0.37 | 1.07 | 80.33 | 1.01 |
| **Group-Level Effects** | | | | | | |
| Subject random effect (Intercept) | 1.08 | 1.07 | 0.93 | 1.23 | 200 | 1 |
| Stimulus random effect (Intercept) | 0.09 | 0.08 | 0 | 0.18 | 0.15 | 1 |

1. **Block model:** Accuracy ~ Block + (1|Id) + (1|Stim)

|  | | | **95% HDI** | |  | |
| --- | --- | --- | --- | --- | --- | --- |
| **Parameter** | **Mean** | **MAP** | **Lower** | **Upper** | **BF** | **Rhat** |
| Intercept | 3 | 3 | 2.69 | 3.32 | 3.93E+13 | 1 |
| **Block: Main Effect** | | | | | | |
| Block Tr2 | 0.9 | 0.86 | 0.6 | 1.23 | 6.17E+02 | 1 |
| Block Tr3 | 1.48 | 1.48 | 1.1 | 1.85 | 3.97E+05 | 1 |
| Block Ts1 | -3.04 | -3.05 | -3.26 | -2.82 | 1.23E+54 | 1 |
| Block Ts2 | -2.15 | -2.14 | -2.36 | -1.91 | 1.35E+16 | 1 |
| Block Ts3 | -1.72 | -1.73 | -1.94 | -1.48 | 4.11E+15 | 1 |
| Block Ts4 | -1.73 | -1.72 | -1.97 | -1.51 | 3.37E+11 | 1 |
| **Group-Level Effects** | | | | | | |
| Subject random effect (Intercept) | 1.46 | 1.45 | 1.28 | 1.67 | 2.18E+18 | 1.01 |
| Stimulus random effect (Intercept) | 0.14 | 0.12 | 0.04 | 0.26 | 1.18E+00 | 1 |

1. **Complete LOO-CV output.**

| **Model** | **elpd_diff** | **se_diff** | **elpd_loo** | **se_elpd_loo** | **p_loo** | **se_p_loo** | **looic** | **se_looic** |
| --- | --- | --- | --- | --- | --- | --- | --- | --- |
| Block x Group x Reactivation type | 0 | 0 | -3714.938 | 61.764 | 157.895 | 3.751 | 7429.875 | 123.528 |
| Block + Group + Reactivation type | -56.448 | 13.853 | -3771.385 | 63.18 | 125.933 | 2.67 | 7542.77 | 126.36 |
| Group + Block | -58.497 | 13.84 | -3773.435 | 63.041 | 132.332 | 2.849 | 7546.869 | 126.082 |
| Block | -61.901 | 14.216 | -3776.838 | 63.155 | 141.923 | 3.213 | 7553.676 | 126.311 |
| Group | -1192.768 | 44.562 | -4907.705 | 54.833 | 122.718 | 2.26 | 9815.411 | 109.665 |
| Reactivation type | -1197.58 | 44.418 | -4912.518 | 54.848 | 134.669 | 2.644 | 9825.035 | 109.697 |
| Intercept | -1199.713 | 44.552 | -4914.65 | 54.823 | 131.06 | 2.617 | 9829.301 | 109.647 |
|  |  |  |  |  |  |  |  |  |

1. **Item Memory (Free Recall) posterior models estimates and full LOO-CV analysis.**

Results of generalized linear mixed effects model for memory accuracy (note: coefficients on the log-odds scale). Posterior mean Estimate, Maximum a Posteriori Probability (MAP),95% highest density interval (HDI), Bayes Factor (BF) and Rhat diagnostic**.**

1. **Null model**: Recall ~ 1 + (1|Id)

|  | | | **95% HDI** | | |  | | |  |
| --- | --- | --- | --- | --- | --- | --- | --- | --- | --- |
| **Parameter** | **Mean** | **MAP** | | **Lower** | **Upper** | | **BF** | **Rhat** | |
| Intercept | 1.51 | 1.51 | | 1.37 | 1.66 | | 7.91E+18 | 1 | |
| Subject random effect (Intercept) | 0.3 | 0.36 | | 0.01 | 0.54 | | 5.00E-01 | 1 | |

1. **Interaction Model (Reported):** Recall ~ Group * Reactivation type + (1|Id) + (1|Stim)

|  | | | **95% HDI** | |  | |
| --- | --- | --- | --- | --- | --- | --- |
| **Parameter** | **Mean** | **MAP** | **Lower** | **Upper** | **BF** | **Rhat** |
| Intercept | 0.56 | 0.55 | 0.26 | 0.87 | 2.47 | 1 |
| **Group: Main Effect** | | | | | | |
| Group Older Adults | 1.08 | 1.04 | 0.7 | 1.54 | 11467.4 | 1 |
| Group Younger Adults | 1.57 | 1.55 | 1.11 | 2.02 | 154353 | 1 |
| **Reactivation type: Main Effect** | | | | | | |
| Reactivation type | 0.73 | 0.75 | 0.36 | 1.13 | 23.55 | 1 |
| **Group x Reactivation type: Interaction** | | | | | | |
| Group Older Adults x Reactivation type | -0.83 | -0.8 | -1.45 | -0.26 | 3.7 | 1 |
| Group Younger Adults x Reactivation type | -0.32 | -0.35 | -0.98 | 0.42 | 0.17 | 1 |
| **Group-Level Effects** | | | | | | |
| Subject random effect (Intercept) | 0.09 | 0.04 | 0 | 0.21 | 0.04 | 1 |
| Stimulus random effect (Intercept) | 0.22 | 0.19 | 0 | 0.43 | 1.59 | 1 |

1. **Main effects Model:** Recall ~ Group + Reactivation type + (1|Id) + (1|Stim)

|  | | | **95% HDI** | |  | |
| --- | --- | --- | --- | --- | --- | --- |
| **Parameter** | **Mean** | **MAP** | **Lower** | **Upper** | **BF** | **Rhat** |
| Intercept | 0.71 | 0.7 | 0.44 | 0.98 | 66.45 | 1 |
| **Group: Main Effect** | | | | | | |
| Group Older Adults | 0.7 | 0.71 | 0.41 | 1 | 168.09 | 1 |
| Group Younger Adults | 1.43 | 1.44 | 1.09 | 1.78 | 568352.89 | 1 |
| **Reactivation type: Main Effect** | | | | | | |
| Reactivation type | 0.4 | 0.37 | 0.12 | 0.68 | 2.07 | 1 |
| **Group-Level Effects** | | | | | | |
| Subject random effect (Intercept) | 0.09 | 0.02 | 0 | 0.23 | 0.04 | 1 |
| Stimulus random effect (Intercept) | 0.22 | 0.19 | 0 | 0.44 | 0.26 | 1 |

1. **Group model:** Recall ~ Group + (1|Id) + (1|Stim)

|  | | | **95% HDI** | |  | |
| --- | --- | --- | --- | --- | --- | --- |
| **Parameter** | **Mean** | **MAP** | **Lower** | **Upper** | **BF** | **Rhat** |
| Intercept | 0.89 | 0.88 | 0.63 | 1.13 | 1246.31 | 1 |
| **Group: Main Effect** | | | | | | |
| Group Older Adults | 0.7 | 0.71 | 0.41 | 1.02 | 462.94 | 1 |
| Group Younger Adults | 1.41 | 1.4 | 1.05 | 1.76 | 2652673.12 | 1 |
| **Group-Level Effects** | | | | | | |
| Subject random effect (Intercept) | 0.11 | 0.04 | 0 | 0.25 | 0.07 | 1 |
| Stimulus random effect (Intercept) | 0.21 | 0.2 | 0 | 0.44 | 0.28 | 1 |

1. **Complete LOO-CV output.**

| **Model** | **elpd_diff** | **se_diff** | **elpd_loo** | **se_elpd_loo** | **p_loo** | **se_p_loo** | **looic** | **se_looic** |
| --- | --- | --- | --- | --- | --- | --- | --- | --- |
| Group x Reactivation type | 0 | 0 | -684.031 | 22.739 | 13.104 | 0.602 | 1368.062 | 45.477 |
| Group + Reactivation type | -2.17 | 2.636 | -686.201 | 22.661 | 11.481 | 0.503 | 1372.402 | 45.323 |
| Group | -5.547 | 3.825 | -689.578 | 22.491 | 11.195 | 0.484 | 1379.156 | 44.982 |
| Intercept | -37.369 | 8.336 | -721.4 | 22.342 | 21.43 | 0.889 | 1442.8 | 44.685 |

1. **Item Memory (Recognition) posterior models estimates and full LOO-CV analysis.**

Results of generalized linear mixed effects model for memory accuracy (note: coefficients on the log-odds scale). Posterior mean Estimate, Maximum a Posteriori Probability (MAP),95% highest density interval (HDI), Bayes Factor (BF) and Rhat diagnostic**.**

1. **Null model:** Recognition ~ 1 + (1|Id)

|  | | | **95% HDI** | |  | |
| --- | --- | --- | --- | --- | --- | --- |
| **Parameter** | **Mean** | **MAP** | **Lower** | **Upper** | **BF** | **Rhat** |
| Intercept | 4.25 | 4.19 | 3.77 | 4.72 | 9.36E+30 | 1 |
| Subject random effect (Intercept) | 1.06 | 1.01 | 0.62 | 1.56 | 9.38E+01 | 1 |

1. **Interaction Model:** Group * Reactivation type * Stimulus type (old/new) + (1|Id) + (1|Stim)

|  | | | | **95% HDI** | | |  | | |
| --- | --- | --- | --- | --- | --- | --- | --- | --- | --- |
| **Parameter** | **Mean** | **MAP** | **Lower** | | **Upper** | **BF** | | **Rhat** |  |
| Intercept | 2.44 | 2.44 | 1.94 | | 2.96 | 1.14E+08 | | 1 |  |
| **Group: Main Effect** | | | | | | | | | |
| Group Older Adults | 2.21 | 2.21 | 1.13 | | 3.43 | 243.44 | | 1 |  |
| Group Younger Adults | 2.6 | 2.48 | 1.28 | | 4.09 | 402.18 | | 1 |  |
| **Reactivation type: Main Effect** | | | | | | | | | |
| Reactivation type | 0.87 | 0.86 | 0.16 | | 1.61 | 2.31 | | 1 |  |
| **Stimulus Type: Main Effect** | | | | | | | | | |
| Stimulus Type | 0.61 | 0.57 | -0.12 | | 1.4 | 0.47 | | 1 |  |
| **Group x Reactivation type: Interaction** | | | | | | | | | |
| Group Older Adults x Reactivation type | 0.2 | 0.02 | -1.73 | | 2.07 | 0.29 | | 1 |  |
| Group Younger Adults x Reactivation type | -0.04 | -0.16 | -1.95 | | 2.54 | 0.35 | | 1 |  |
| **Group x Stimulus Type: Interaction** | | | | | | | | | |
| Group Older Adults x Stimulus Type | 0.4 | 0.22 | -1.5 | | 2.33 | 0.32 | | 1 |  |
| Group Younger Adults x Stimulus Type | 0.14 | 0.08 | -1.84 | | 2.22 | 0.29 | | 1 |  |
| **Reactivation type x Stimulus Type: Interaction** | | | | | | | | | |
| Reactivation type x Stimulus Type | -0.62 | -0.55 | -1.66 | | 0.37 | 0.29 | | 1 |  |
| **Group x Reactivation type x Stimulus Type: Interaction** | | | | | | | | | |
| Group Older Adults x Reactivation type x Stimulus Type | -1.35 | -1.16 | -4.07 | | 1.13 | 0.66 | | 1 |  |
| Group Younger Adults x Reactivation type x Stimulus Type | -0.73 | -0.67 | -3.61 | | 2.13 | 0.48 | | 1 |  |
| **Group-Level Effects** | | | | | | | | | |
| Subject random effect (Intercept) | 0.3 | 0.05 | 0 | | 0.69 | 0.09 | | 1 |  |
| Stimulus random effect (Intercept) | 0.29 | 0.22 | 0 | | 0.66 | 0.12 | | 1 |  |

1. **Main effects Model:** Recognition ~Group + Reactivation type + Stimulus type (old/new) + (1|Id) + (1|Stim)

|  | | | **95% HDI** | |  | |
| --- | --- | --- | --- | --- | --- | --- |
| **Parameter** | **Mean** | **MAP** | **Lower** | **Upper** | **BF** | **Rhat** |
| Intercept | 2.6 | 2.56 | 2.08 | 3.11 | 927872535.2 | 1 |
| **Group: Main Effect** | | | | | | |
| Group Older Adults | 2 | 1.95 | 1.23 | 2.76 | 3371.45 | 1 |
| Group Younger Adults | 2.29 | 2.16 | 1.44 | 3.15 | 17190.44 | 1 |
| **Reactivation type: Main Effect** | | | | | | |
| Reactivation type | 0.48 | 0.48 | -0.02 | 1 | 0.52 | 1 |
| **Stimulus Type: Main Effect** | | | | | | |
| Stimulus Type | 0.3 | 0.35 | -0.29 | 0.86 | 0.15 | 1 |
| **Group-Level Effects** | | | | | | |
| Subject random effect (Intercept) | 0.31 | 0.05 | 0 | 0.69 | 0.19 | 1 |
| Stimulus random effect (Intercept) | 0.3 | 0.2 | 0 | 0.66 | 0.08 | 1 |

1. **Group + Stimulus Type Model:** Recognition ~Group + Stimulus type (old/new) + (1|Id) + (1|Stim)

|  | | | **95% HDI** | | |  | | |  |
| --- | --- | --- | --- | --- | --- | --- | --- | --- | --- |
| **Parameter** | **Mean** | **MAP** | | **Lower** | **Upper** | | **BF** | **Rhat** | |
| Intercept | 2.82 | 2.81 | | 2.37 | 3.3 | | 1.14E+10 | 1 | |
| **Group: Main Effect** | | | | | | | | |  |
| Group Older Adults | 2 | 1.99 | | 1.23 | 2.75 | | 2.51E+03 | 1 | |
| Group Younger Adults | 2.29 | 2.17 | | 1.45 | 3.19 | | 6.47E+04 | 1 | |
| **Stimulus Type: Main Effect** | | | | | | | | |  |
| Stimulus Type | 0.3 | 0.32 | | -0.28 | 0.88 | | 1.50E-01 | 1 | |
| **Group-Level Effects** | | | | | | | | |  |
| Subject random effect (Intercept) | 0.33 | 0.18 | | 0 | 0.72 | | 2.70E-01 | 1 | |
| Stimulus random effect (Intercept) | 0.3 | 0.22 | | 0 | 0.64 | | 2.10E-01 | 1 | |

1. **Group Model:** Recognition ~Group + (1|Id) + (1|Stim)

|  | | | **95% HDI** | | |  | | |  |
| --- | --- | --- | --- | --- | --- | --- | --- | --- | --- |
| **Parameter** | **Mean** | **MAP** | | **Lower** | **Upper** | | **BF** | **Rhat** | |
| Intercept | 2.96 | 2.92 | | 2.59 | 3.35 | | 2.70E+20 | 1 | |
| **Group: Main Effect** | | | | | | | | |  |
| Group Older Adults | 1.99 | 2.01 | | 1.24 | 2.77 | | 6.38E+03 | 1 | |
| Group Younger Adults | 2.28 | 2.31 | | 1.44 | 3.16 | | 4.45E+03 | 1 | |
| **Group-Level Effects** | | | | | | | | |  |
| Subject random effect (Intercept) | 0.34 | 0.23 | | 0 | 0.73 | | 4.80E-01 | 1 | |
| Stimulus random effect (Intercept) | 0.29 | 0.24 | | 0 | 0.62 | | 1.80E-01 | 1 | |

1. **Complete LOO-CV output.**

| **Model** | **elpd_diff** | **se_diff** | **elpd_loo** | **se_elpd_loo** | **p_loo** | **se_p_loo** | **looic** | **se_looic** |
| --- | --- | --- | --- | --- | --- | --- | --- | --- |
| Group + Reactivation type + Stimulus Type | 0 | 0 | -302.596 | 28.594 | 16.737 | 1.983 | 605.192 | 57.187 |
| Group | -0.694 | 2.136 | -303.29 | 28.508 | 16.737 | 1.985 | 606.58 | 57.016 |
| Group + Stimulus Type | -0.901 | 1.738 | -303.497 | 28.62 | 17.668 | 2.093 | 606.994 | 57.239 |
| Group x Reactivation type x Stimulus Type | -4.247 | 2.696 | -306.843 | 29.434 | 23.279 | 3.346 | 613.687 | 58.868 |
| Intercept | -19.687 | 6.5 | -322.283 | 30.023 | 40.499 | 4.692 | 644.567 | 60.046 |

1. **Prior sensitivity analysis:**

To estimate the prior´s influence on the posterior estimates, we: **1)** refitted all models with different priors and performed a Leave-one-out cross-validation for model comparison (including all predictors against reduced models without the predictor of interest) , **2)** Conducted a Leave-one-out cross-validation between winning models with different priors.

We found that the best performing models using different priors were the same as those reported in the main manuscript (a- ELPD differences). These models have similar prediction accuracy irrespectively of the prior specification (b – ELPD differences). In sum, prior specification had a small influence on the reported results.

- **Associative Memory (face-name pairs).**

1. **LOO-CV for each model.**

| **Model´s Prior** | **elpd_diff** | **se_diff** |
| --- | --- | --- |
| **Generic Weakly Prior (normal(0, 1))** |  |  |
| Block * Group * Reactivation type | 0 | 0 |
| Block + Group + Reactivation type | -56.49 | 12.53 |
| Group + Block | -58.54 | 12.53 |
| Block | -61.94 | 12.94 |
| Group | -1192.81 | 43.42 |
| Reactivation type | -1197.62 | 43.27 |
| Intercept | -1194.44 | 43.4 |
|  |  |  |
| **Generic Weakly Informative Prior (T-Student (7,0, 2.5)** |  |  |
| Block * Group * Reactivation type | 0 | 0 |
| Block + Group + Reactivation type | -57.59 | 12.16 |
| Group + Block | -59.77 | 11.33 |
| Block | -62.04 | 11.48 |
| Group | -1189.31 | 44.87 |
| Reactivation type | -1190.44 | 44.2 |
| Intercept | -1192.03 | 44.15 |
|  |  |  |
| **Weakly Informative Prior (T-Student (7,0, 2.5))** |  |  |
| Block * Group * Reactivation type | 0 | 0 |
| Block + Group + Reactivation type | -55.1 | 13.96 |
| Group + Block | -57.14 | 13.94 |
| Block | -60.55 | 14.31 |
| Group | -1191.42 | 44.62 |
| Reactivation type | -1196.23 | 44.47 |
| Intercept | -1198.36 | 44.61 |

1. **Model Comparison**

**
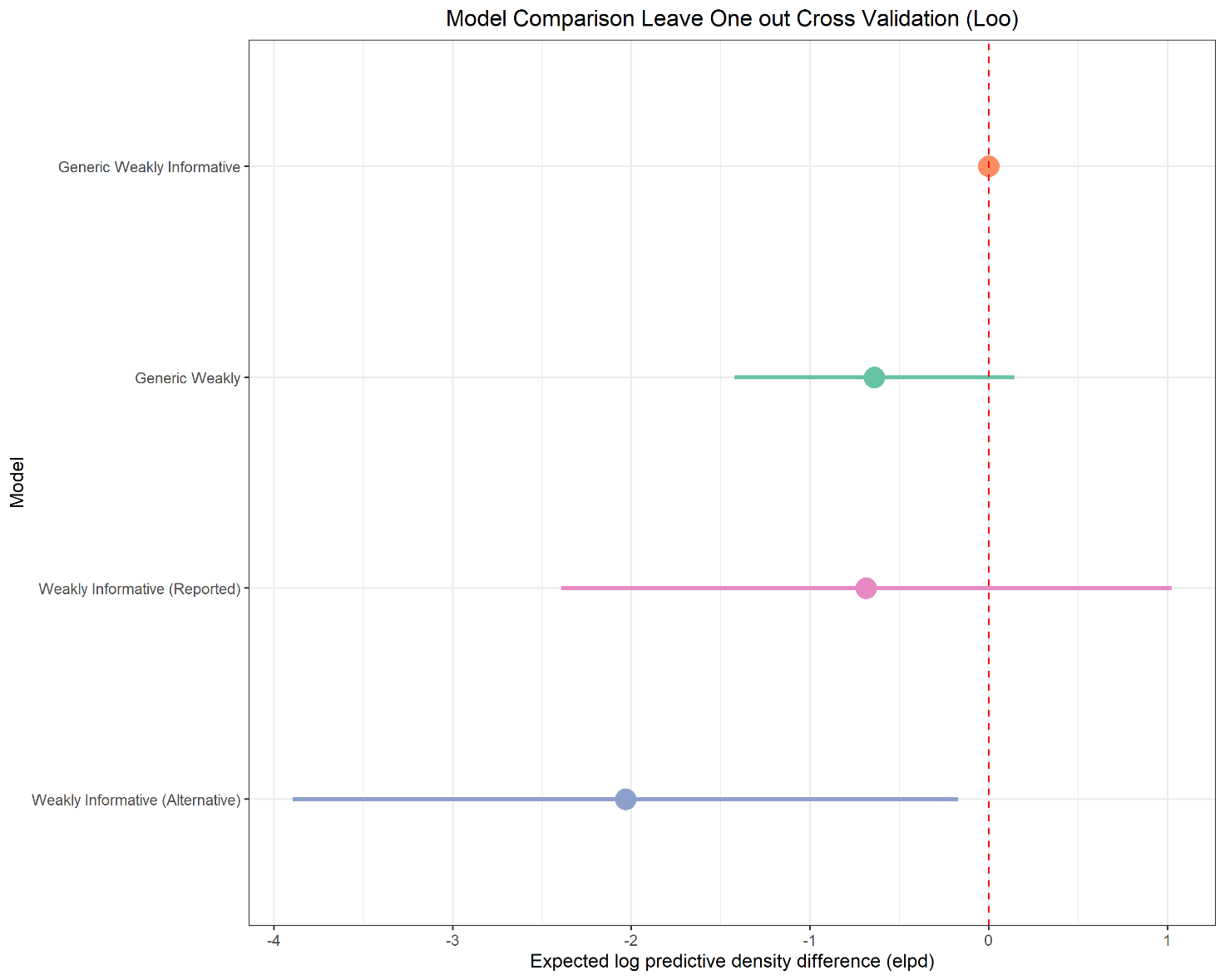
**

- **Item Memory (Free- Recall)**

1. **LOO-CV for each model.**

| **Model´s Prior** | **elpd_diff** | **se_diff** |
| --- | --- | --- |
| **Generic Weakly Prior (normal(0, 1))** |  |  |
| Group * Reactivation type | 0 | 0 |
| Group + Reactivation type | -1.94 | 2.22 |
| Group | -5.41 | 3.47 |
| Intercept | -36.2 | 8 |
|  |  |  |
| **Generic Weakly Informative Prior (T-Student (7,0, 2.5)** |  |  |
| Group * Reactivation type | 0 | 0 |
| Group + Reactivation type | -1.85 | 2.27 |
| Group | -5.22 | 3.54 |
| Intercept | -37.05 | 7.99 |
|  |  |  |
| **Weakly Informative Prior (T-Student (7,0, 2.5))** |  |  |
| Group * Reactivation type | 0 | 0 |
| Group + Reactivation type | -1.89 | 2.67 |
| Group | -5.27 | 3.89 |
| Intercept | -37.09 | 8.41 |

1. **Model Comparison**

**
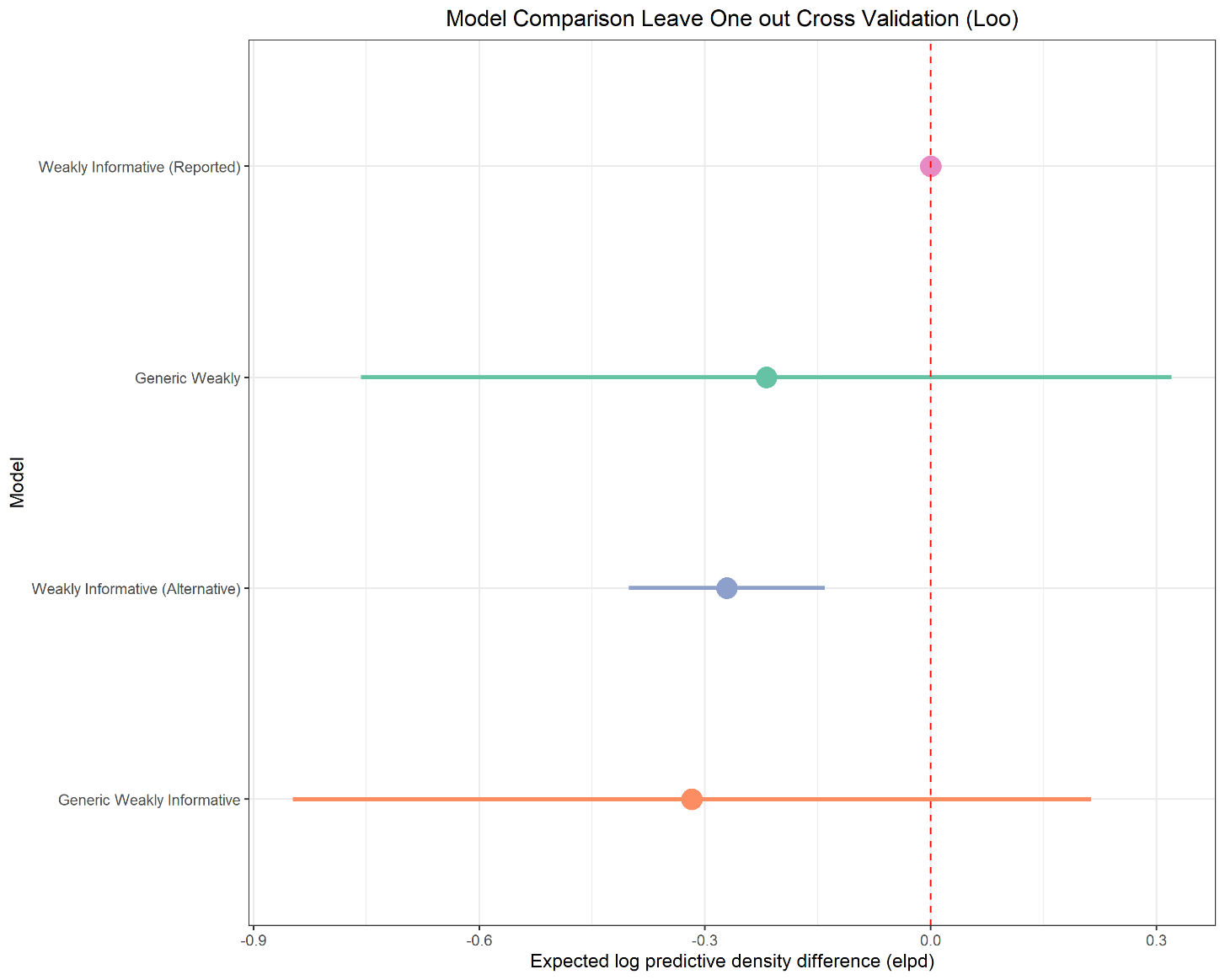
**

- **Item Memory (Recognition)**

1. **LOO-CV for each model.**

| **Model´s Prior** | **elpd_diff** | **se_diff** |
| --- | --- | --- |
| **Generic Weakly Prior** |  |  |
| Group + Reactivation type+ Stimulus type | 0 | 0 |
| Group | -0.67 | 2.14 |
| Group + Stimulus Type | -0.9 | 1.76 |
| Group * Reactivation type* Stimulus type | -1.35 | 1.74 |
| Intercept | -19.41 | 6.5 |
|  |  |  |
| **Generic Weakly Informative Prior** |  |  |
| Group + Reactivation type+ Stimulus type | 0 | 0 |
| Group | -0.69 | 2.1 |
| Group + Stimulus Type | -0.89 | 1.63 |
| Group * Reactivation type* Stimulus type | -1.73 | 1.84 |
| Intercept | -20.1 | 6.1 |
|  |  |  |
| **Weakly Informative Prior** |  |  |
| Group + Reactivation type+ Stimulus type | 0 | 0 |
| Group | -0.69 | 2.13 |
| Group + Stimulus Type | -0.9 | 1.73 |
| Group * Reactivation type* Stimulus type | -3.84 | 2.59 |
| Intercept | -19.68 | 6.4 |

1. **Model Comparison**

**
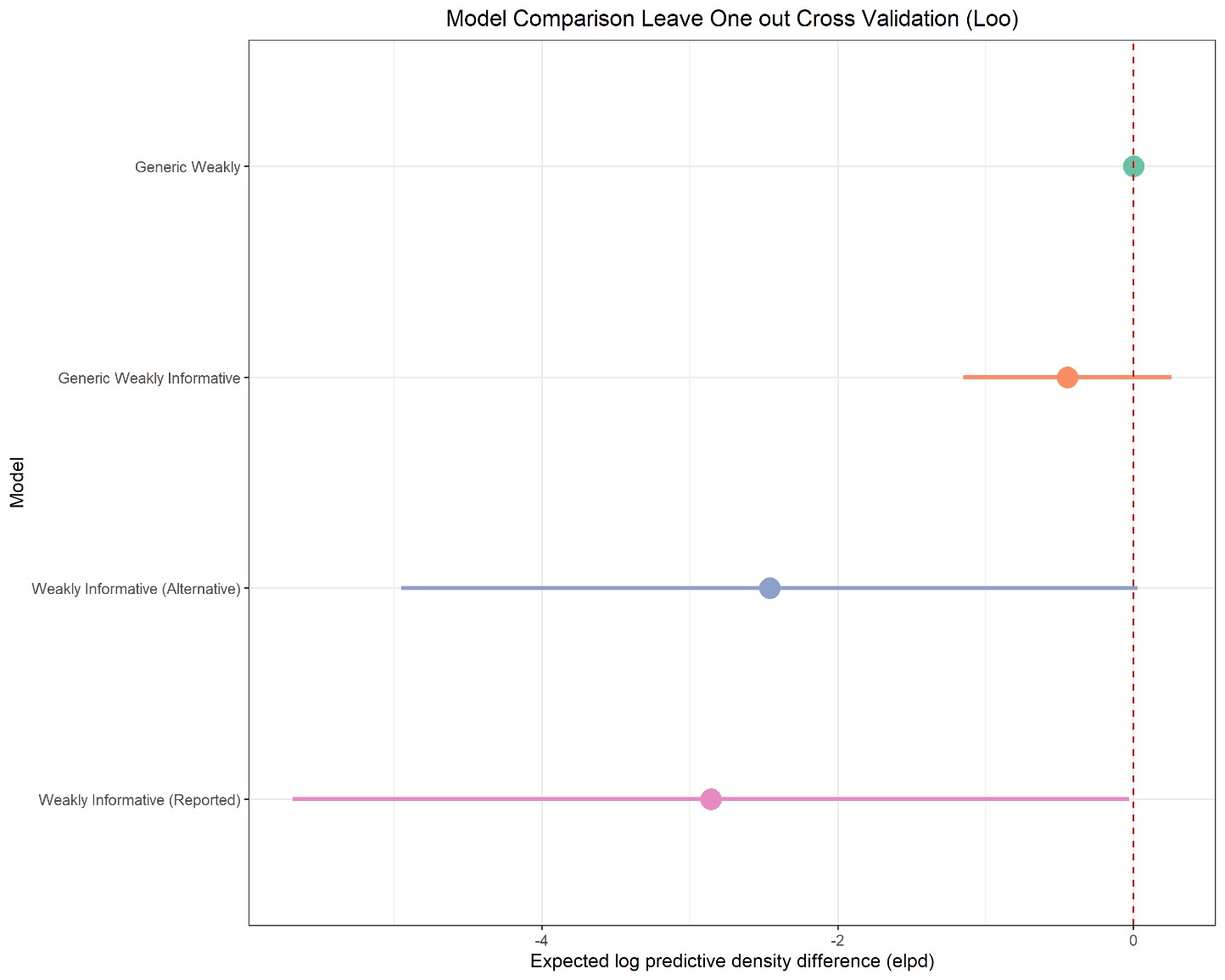
**

1. **Additional Measures.**

**Descriptive statistics table.**
